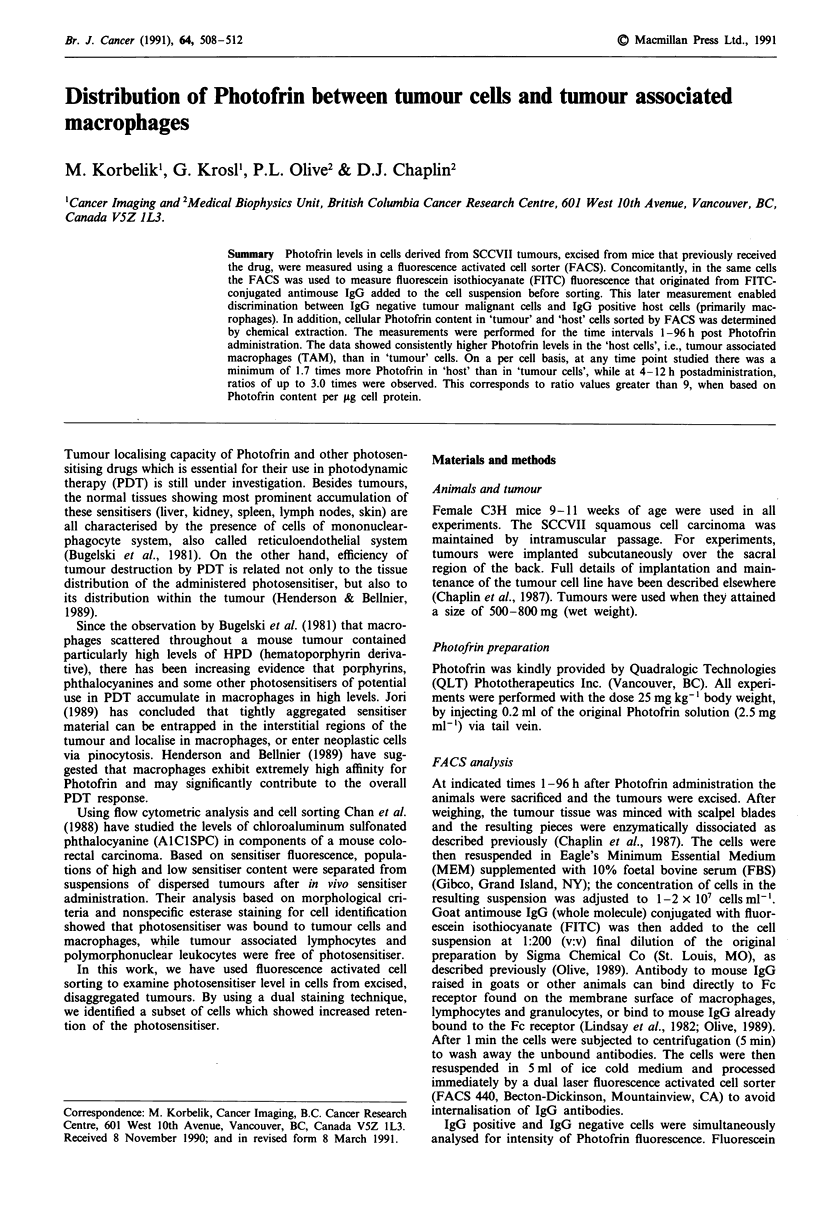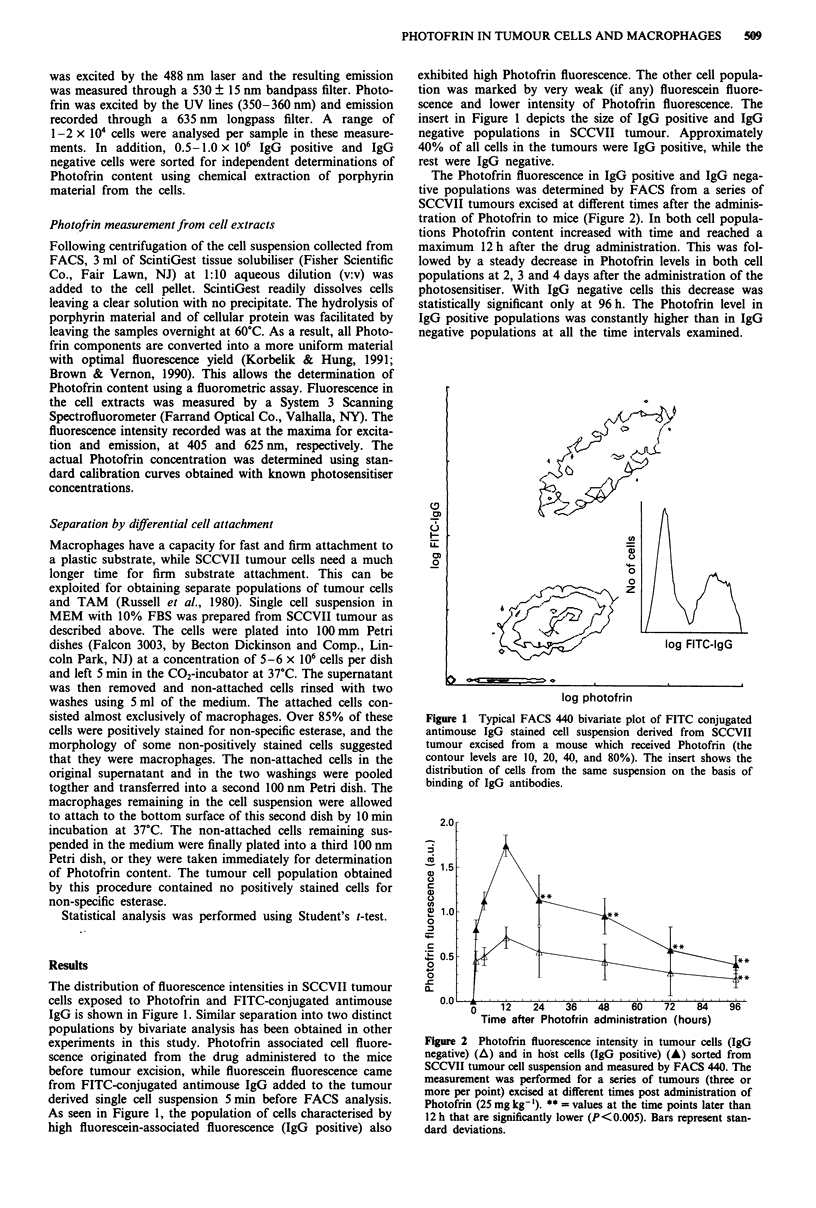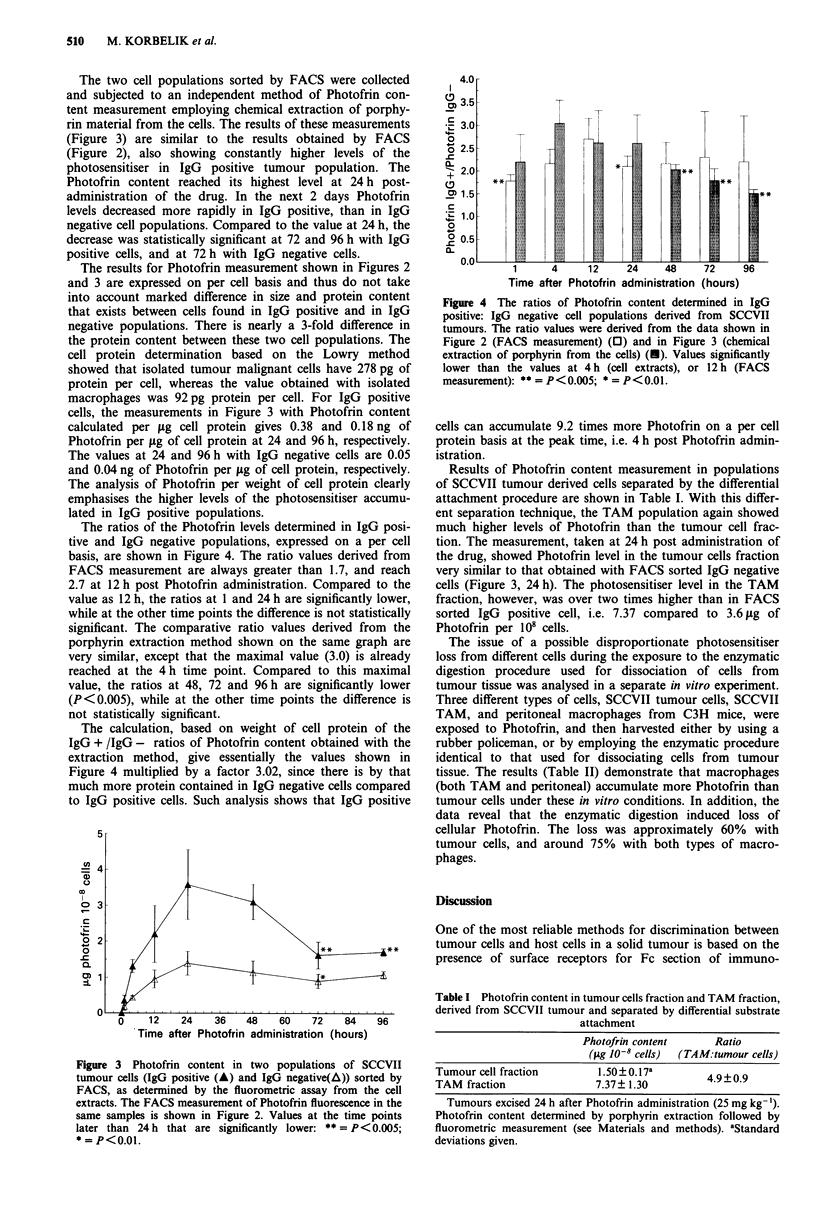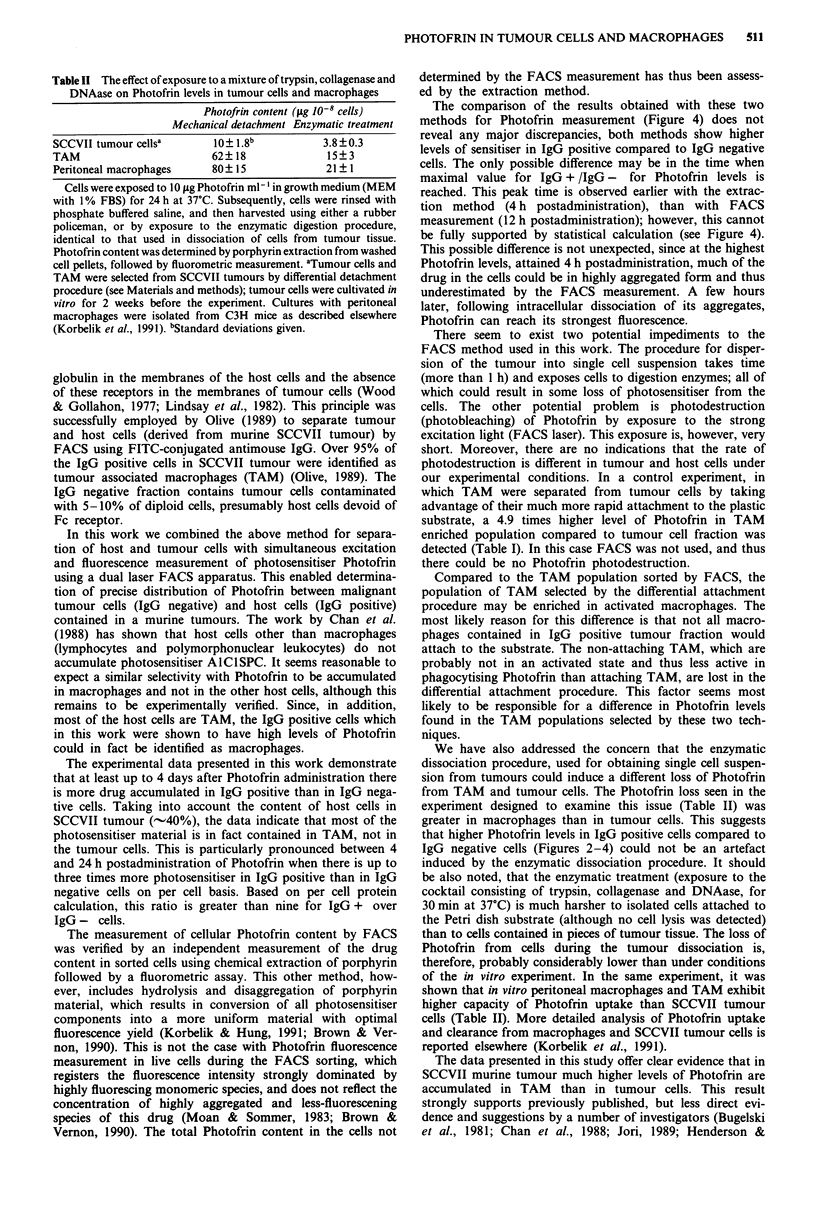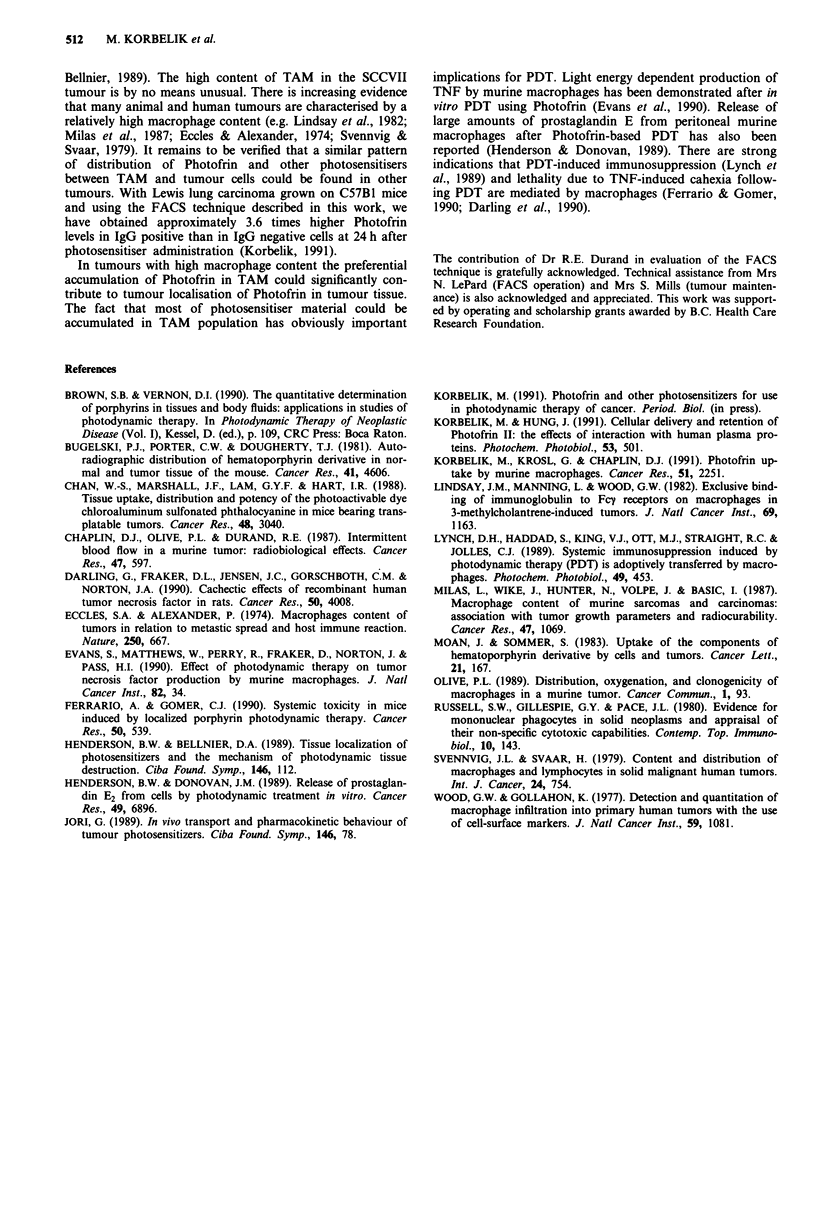# Distribution of Photofrin between tumour cells and tumour associated macrophages.

**DOI:** 10.1038/bjc.1991.339

**Published:** 1991-09

**Authors:** M. Korbelik, G. Krosl, P. L. Olive, D. J. Chaplin

**Affiliations:** Cancer Imaging Unit, British Columbia Cancer Research Centre, Vancouver, Canada.

## Abstract

Photofrin levels in cells derived from SCCVII tumours, excised from mice that previously received the drug, were measured using a fluorescence activated cell sorter (FACS). Concomitantly, in the same cells the FACS was used to measure fluorescein isothiocyanate (FITC) fluorescence that originated from FITC-conjugated antimouse IgG added to the cell suspension before sorting. This later measurement enabled discrimination between IgG negative tumour malignant cells and IgG positive host cells (primarily macrophages). In addition, cellular Photofrin content in 'tumour' and 'host' cells sorted by FACS was determined by chemical extraction. The measurements were performed for the time intervals 1-96 h post Photofrin administration. The data showed consistently higher Photofrin levels in the 'host cells', i.e., tumour associated macrophages (TAM), than in 'tumour' cells. On a per cell basis, at any time point studied there was a minimum of 1.7 times more Photofrin in 'host' than in 'tumour cells', while at 4-12 h postadministration, ratios of up to 3.0 times were observed. This corresponds to ratio values greater than 9, when based on Photofrin content per micrograms cell protein.


Br. J. Cancer (1991), 64, 508-512 © Macmillan Press Ltd., 1991

### Distribution of Photofrin between tumour cells and tumour associated macrophages

M. Korbelik[1], G. Krosl[1], P.L. Olive[2] & D.J. Chaplin[2]

[1]*Cancer Imaging and* [2]*Medical Biophysics Unit, British Columbia Cancer Research Centre, 601 West 10th Avenue, Vancouver, BC, Canada V5Z 1L3.*

**Summary** Photofrin levels in cells derived from SCCVII tumours, excised from mice that previously received the drug, were measured using a fluorescence activated cell sorter (FACS). Concomitantly, in the same cells the FACS was used to measure fluorescein isothiocyanate (FITC) fluorescence that originated from FITC-conjugated antimouse IgG added to the cell suspension before sorting. This later measurement enabled discrimination between IgG negative tumour malignant cells and IgG positive host cells (primarily macrophages). In addition, cellular Photofrin content in 'tumour' and 'host' cells sorted by FACS was determined by chemical extraction. The measurements were performed for the time intervals 1-96 h post Photofrin administration. The data showed consistently higher Photofrin levels in the 'host cells', i.e., tumour associated macrophages (TAM), than in 'tumour' cells. On a per cell basis, at any time point studied there was a minimum of 1.7 times more Photofrin in 'host' than in 'tumour cells', while at 4-12 h postadministration, ratios of up to 3.0 times were observed. This corresponds to ratio values greater than 9, when based on Photofrin content per μg cell protein.

Tumour localising capacity of Photofrin and other photosensitising drugs which is essential for their use in photodynamic therapy (PDT) is still under investigation. Besides tumours, the normal tissues showing most prominent accumulation of these sensitisers (liver, kidney, spleen, lymph nodes, skin) are all characterised by the presence of cells of mononuclear-phagocyte system, also called reticuloendothelial system (Bugelski *et al.*, 1981). On the other hand, efficiency of tumour destruction by PDT is related not only to the tissue distribution of the administered photosensitiser, but also to its distribution within the tumour (Henderson & Bellnier, 1989).

Since the observation by Bugelski *et al.* (1981) that macrophages scattered throughout a mouse tumour contained particularly high levels of HPD (hematoporphyrin derivative), there has been increasing evidence that porphyrins, phthalocyanines and some other photosensitisers of potential use in PDT accumulate in macrophages in high levels. Jori (1989) has concluded that tightly aggregated sensitiser material can be entrapped in the interstitial regions of the tumour and localise in macrophages, or enter neoplastic cells via pinocytosis. Henderson and Bellnier (1989) have suggested that macrophages exhibit extremely high affinity for Photofrin and may significantly contribute to the overall PDT response.

Using flow cytometric analysis and cell sorting Chan *et al.* (1988) have studied the levels of chloroaluminum sulfonated phthalocyanine (A1C1SPC) in components of a mouse colorectal carcinoma. Based on sensitiser fluorescence, populations of high and low sensitiser content were separated from suspensions of dispersed tumours after *in vivo* sensitiser administration. Their analysis based on morphological criteria and nonspecific esterase staining for cell identification showed that photosensitiser was bound to tumour cells and macrophages, while tumour associated lymphocytes and polymorphonuclear leukocytes were free of photosensitiser.

In this work, we have used fluorescence activated cell sorting to examine photosensitiser level in cells from excised, disaggregated tumours. By using a dual staining technique, we identified a subset of cells which showed increased retention of the photosensitiser.

Correspondence: M. Korbelik, Cancer Imaging, B.C. Cancer Research Centre, 601 West 10th Avenue, Vancouver, BC, Canada V5Z 1L3.
Received 8 November 1990; and in revised form 8 March 1991.

### Materials and methods

### *Animals and tumour*

Female C3H mice 9-11 weeks of age were used in all experiments. The SCCVII squamous cell carcinoma was maintained by intramuscular passage. For experiments, tumours were implanted subcutaneously over the sacral region of the back. Full details of implantation and maintenance of the tumour cell line have been described elsewhere (Chaplin *et al.*, 1987). Tumours were used when they attained a size of 500-800 mg (wet weight).

### *Photofrin preparation*

Photofrin was kindly provided by Quadralogic Technologies (QLT) Phototherapeutics Inc. (Vancouver, BC). All experiments were performed with the dose 25 mg $kg^{-1}$ body weight, by injecting 0.2 ml of the original Photofrin solution (2.5 mg $ml^{-1}$) via tail vein.

### *FACS analysis*

At indicated times 1-96 h after Photofrin administration the animals were sacrificed and the tumours were excised. After weighing, the tumour tissue was minced with scalpel blades and the resulting pieces were enzymatically dissociated as described previously (Chaplin *et al.*, 1987). The cells were then resuspended in Eagle's Minimum Essential Medium (MEM) supplemented with 10% foetal bovine serum (FBS) (Gibco, Grand Island, NY); the concentration of cells in the resulting suspension was adjusted to $1-2 \times 10^7$ cells $ml^{-1}$. Goat antimouse IgG (whole molecule) conjugated with fluorescein isothiocyanate (FITC) was then added to the cell suspension at 1:200 (v:v) final dilution of the original preparation by Sigma Chemical Co (St. Louis, MO), as described previously (Olive, 1989). Antibody to mouse IgG raised in goats or other animals can bind directly to Fc receptor found on the membrane surface of macrophages, lymphocytes and granulocytes, or bind to mouse IgG already bound to the Fc receptor (Lindsay *et al.*, 1982; Olive, 1989). After 1 min the cells were subjected to centrifugation (5 min) to wash away the unbound antibodies. The cells were then resuspended in 5 ml of ice cold medium and processed immediately by a dual laser fluorescence activated cell sorter (FACS 440, Becton-Dickinson, Mountainview, CA) to avoid internalisation of IgG antibodies.

IgG positive and IgG negative cells were simultaneously analysed for intensity of Photofrin fluorescence. Fluorescein was excited by the 488 nm laser and the resulting emission was measured through a 530 ± 15 nm bandpass filter. Photofrin was excited by the UV lines (350–360 nm) and emission recorded through a 635 nm longpass filter. A range of $1-2 \times 10^4$ cells were analysed per sample in these measurements. In addition, $0.5-1.0 \times 10^6$ IgG positive and IgG negative cells were sorted for independent determinations of Photofrin content using chemical extraction of porphyrin material from the cells.

*Photofrin measurement from cell extracts*

Following centrifugation of the cell suspension collected from FACS, 3 ml of ScintiGest tissue solubiliser (Fisher Scientific Co., Fair Lawn, NJ) at 1:10 aqueous dilution (v:v) was added to the cell pellet. ScintiGest readily dissolves cells leaving a clear solution with no precipitate. The hydrolysis of porphyrin material and of cellular protein was facilitated by leaving the samples overnight at 60°C. As a result, all Photofrin components are converted into a more uniform material with optimal fluorescence yield (Korbelik & Hung, 1991; Brown & Vernon, 1990). This allows the determination of Photofrin content using a fluorometric assay. Fluorescence in the cell extracts was measured by a System 3 Scanning Spectrofluorometer (Farrand Optical Co., Valhalla, NY). The fluorescence intensity recorded was at the maxima for excitation and emission, at 405 and 625 nm, respectively. The actual Photofrin concentration was determined using standard calibration curves obtained with known photosensitiser concentrations.

*Separation by differential cell attachment*

Macrophages have a capacity for fast and firm attachment to a plastic substrate, while SCCVII tumour cells need a much longer time for firm substrate attachment. This can be exploited for obtaining separate populations of tumour cells and TAM (Russell *et al.*, 1980). Single cell suspension in MEM with 10% FBS was prepared from SCCVII tumour as described above. The cells were plated into 100 mm Petri dishes (Falcon 3003, by Becton Dickinson and Comp., Lincoln Park, NJ) at a concentration of $5-6 \times 10^6$ cells per dish and left 5 min in the $CO_2$-incubator at 37°C. The supernatant was then removed and non-attached cells rinsed with two washes using 5 ml of the medium. The attached cells consisted almost exclusively of macrophages. Over 85% of these cells were positively stained for non-specific esterase, and the morphology of some non-positively stained cells suggested that they were macrophages. The non-attached cells in the original supernatant and in the two washings were pooled togther and transferred into a second 100 nm Petri dish. The macrophages remaining in the cell suspension were allowed to attach to the bottom surface of this second dish by 10 min incubation at 37°C. The non-attached cells remaining suspended in the medium were finally plated into a third 100 nm Petri dish, or they were taken immediately for determination of Photofrin content. The tumour cell population obtained by this procedure contained no positively stained cells for non-specific esterase.

Statistical analysis was performed using Student's *t*-test.

### Results

The distribution of fluorescence intensities in SCCVII tumour cells exposed to Photofrin and FITC-conjugated antimouse IgG is shown in Figure 1. Similar separation into two distinct populations by bivariate analysis has been obtained in other experiments in this study. Photofrin associated cell fluorescence originated from the drug administered to the mice before tumour excision, while fluorescein fluorescence came from FITC-conjugated antimouse IgG added to the tumour derived single cell suspension 5 min before FACS analysis. As seen in Figure 1, the population of cells characterised by high fluorescein-associated fluorescence (IgG positive) also exhibited high Photofrin fluorescence. The other cell population was marked by very weak (if any) fluorescein fluorescence and lower intensity of Photofrin fluorescence. The insert in Figure 1 depicts the size of IgG positive and IgG negative populations in SCCVII tumour. Approximately 40% of all cells in the tumours were IgG positive, while the rest were IgG negative.

The Photofrin fluorescence in IgG positive and IgG negative populations was determined by FACS from a series of SCCVII tumours excised at different times after the administration of Photofrin to mice (Figure 2). In both cell populations Photofrin content increased with time and reached a maximum 12 h after the drug administration. This was followed by a steady decrease in Photofrin levels in both cell populations at 2, 3 and 4 days after the administration of the photosensitiser. With IgG negative cells this decrease was statistically significant only at 96 h. The Photofrin level in IgG positive populations was constantly higher than in IgG negative populations at all the time intervals examined.

**Figure 1** Typical FACS 440 bivariate plot of FITC conjugated antimouse IgG stained cell suspension derived from SCCVII tumour excised from a mouse which received Photofrin (the contour levels are 10, 20, 40, and 80%). The insert shows the distribution of cells from the same suspension on the basis of binding of IgG antibodies.

**Figure 2** Photofrin fluorescence intensity in tumour cells (IgG negative) (Δ) and in host cells (IgG positive) (▲) sorted from SCCVII tumour cell suspension and measured by FACS 440. The measurement was performed for a series of tumours (three or more per point) excised at different times post administration of Photofrin (25 mg $kg^{-1}$). ** = values at the time points later than 12 h that are significantly lower ($P<0.005$). Bars represent standard deviations.

The two cell populations sorted by FACS were collected and subjected to an independent method of Photofrin content measurement employing chemical extraction of porphyrin material from the cells. The results of these measurements (Figure 3) are similar to the results obtained by FACS (Figure 2), also showing constantly higher levels of the photosensitiser in IgG positive tumour population. The Photofrin content reached its highest level at 24 h post-administration of the drug. In the next 2 days Photofrin levels decreased more rapidly in IgG positive, than in IgG negative cell populations. Compared to the value at 24 h, the decrease was statistically significant at 72 and 96 h with IgG positive cells, and at 72 h with IgG negative cells.

The results for Photofrin measurement shown in Figures 2 and 3 are expressed on per cell basis and thus do not take into account marked difference in size and protein content that exists between cells found in IgG positive and in IgG negative populations. There is nearly a 3-fold difference in the protein content between these two cell populations. The cell protein determination based on the Lowry method showed that isolated tumour malignant cells have 278 pg of protein per cell, whereas the value obtained with isolated macrophages was 92 pg protein per cell. For IgG positive cells, the measurements in Figure 3 with Photofrin content calculated per µg cell protein gives 0.38 and 0.18 ng of Photofrin per µg of cell protein at 24 and 96 h, respectively. The values at 24 and 96 h with IgG negative cells are 0.05 and 0.04 ng of Photofrin per µg of cell protein, respectively. The analysis of Photofrin per weight of cell protein clearly emphasises the higher levels of the photosensitiser accumulated in IgG positive populations.

The ratios of the Photofrin levels determined in IgG positive and IgG negative populations, expressed on a per cell basis, are shown in Figure 4. The ratio values derived from FACS measurement are always greater than 1.7, and reach 2.7 at 12 h post Photofrin administration. Compared to the value as 12 h, the ratios at 1 and 24 h are significantly lower, while at the other time points the difference is not statistically significant. The comparative ratio values derived from the porphyrin extraction method shown on the same graph are very similar, except that the maximal value (3.0) is already reached at the 4 h time point. Compared to this maximal value, the ratios at 48, 72 and 96 h are significantly lower ($P < 0.005$), while at the other time points the difference is not statistically significant.

The calculation, based on weight of cell protein of the IgG + /IgG – ratios of Photofrin content obtained with the extraction method, give essentially the values shown in Figure 4 multiplied by a factor 3.02, since there is by that much more protein contained in IgG negative cells compared to IgG positive cells. Such analysis shows that IgG positive

**Figure 3** Photofrin content in two populations of SCCVII tumour cells (IgG positive (▲) and IgG negative(Δ)) sorted by FACS, as determined by the fluorometric assay from the cell extracts. The FACS measurement of Photofrin fluorescence in the same samples is shown in Figure 2. Values at the time points later than 24 h that are significantly lower: ** = $P < 0.005$; * = $P < 0.01$.

**Figure 4** The ratios of Photofrin content determined in IgG positive: IgG negative cell populations derived from SCCVII tumours. The ratio values were derived from the data shown in Figure 2 (FACS measurement) (□) and in Figure 3 (chemical extraction of porphyrin from the cells) (■). Values significantly lower than the values at 4 h (cell extracts), or 12 h (FACS measurement): ** = $P < 0.005$; * = $P < 0.01$.

cells can accumulate 9.2 times more Photofrin on a per cell protein basis at the peak time, i.e. 4 h post Photofrin administration.

Results of Photofrin content measurement in populations of SCCVII tumour derived cells separated by the differential attachment procedure are shown in Table I. With this different separation technique, the TAM population again showed much higher levels of Photofrin than the tumour cell fraction. The measurement, taken at 24 h post administration of the drug, showed Photofrin level in the tumour cells fraction very similar to that obtained with FACS sorted IgG negative cells (Figure 3, 24 h). The photosensitiser level in the TAM fraction, however, was over two times higher than in FACS sorted IgG positive cell, i.e. 7.37 compared to 3.6 µg of Photofrin per $10^8$ cells.

The issue of a possible disproportionate photosensitiser loss from different cells during the exposure to the enzymatic digestion procedure used for dissociation of cells from tumour tissue was analysed in a separate *in vitro* experiment. Three different types of cells, SCCVII tumour cells, SCCVII TAM, and peritoneal macrophages from C3H mice, were exposed to Photofrin, and then harvested either by using a rubber policeman, or by employing the enzymatic procedure identical to that used for dissociating cells from tumour tissue. The results (Table II) demonstrate that macrophages (both TAM and peritoneal) accumulate more Photofrin than tumour cells under these *in vitro* conditions. In addition, the data reveal that the enzymatic digestion induced loss of cellular Photofrin. The loss was approximately 60% with tumour cells, and around 75% with both types of macrophages.

### Discussion

One of the most reliable methods for discrimination between tumour cells and host cells in a solid tumour is based on the presence of surface receptors for Fc section of immuno-

**Table I** Photofrin content in tumour cells fraction and TAM fraction, derived from SCCVII tumour and separated by differential substrate attachment

| | *Photofrin content ($\mu g\ 10^{-8}$ cells)* | *Ratio (TAM:tumour cells)* |
|---|---|---|
| Tumour cell fraction | 1.50±0.17[a] | 4.9±0.9 |
| TAM fraction | 7.37±1.30 | |

Tumours excised 24 h after Photofrin administration (25 mg kg⁻¹). Photofrin content determined by porphyrin extraction followed by fluorometric measurement (see Materials and methods). [a]Standard deviations given.

**Table II** The effect of exposure to a mixture of trypsin, collagenase and DNAase on Photofrin levels in tumour cells and macrophages

| | *Photofrin content (µg $10^{-8}$ cells)* | |
|---|---|---|
| | *Mechanical detachment* | *Enzymatic treatment* |
| SCCVII tumour cells[a] | 10±1.8[b] | 3.8±0.3 |
| TAM | 62±18 | 15±3 |
| Peritoneal macrophages | 80±15 | 21±1 |

Cells were exposed to 10 µg Photofrin $ml^{-1}$ in growth medium (MEM with 1% FBS) for 24 h at 37°C. Subsequently, cells were rinsed with phosphate buffered saline, and then harvested using either a rubber policeman, or by exposure to the enzymatic digestion procedure, identical to that used in dissociation of cells from tumour tissue. Photofrin content was determined by porphyrin extraction from washed cell pellets, followed by fluorometric measurement. [a]Tumour cells and TAM were selected from SCCVII tumours by differential detachment procedure (see Materials and methods); tumour cells were cultivated *in vitro* for 2 weeks before the experiment. Cultures with peritoneal macrophages were isolated from C3H mice as described elsewhere (Korbelik *et al.*, 1991). [b]Standard deviations given.

globulin in the membranes of the host cells and the absence of these receptors in the membranes of tumour cells (Wood & Gollahon, 1977; Lindsay *et al.*, 1982). This principle was successfully employed by Olive (1989) to separate tumour and host cells (derived from murine SCCVII tumour) by FACS using FITC-conjugated antimouse IgG. Over 95% of the IgG positive cells in SCCVII tumour were identified as tumour associated macrophages (TAM) (Olive, 1989). The IgG negative fraction contains tumour cells contaminated with 5–10% of diploid cells, presumably host cells devoid of Fc receptor.

In this work we combined the above method for separation of host and tumour cells with simultaneous excitation and fluorescence measurement of photosensitiser Photofrin using a dual laser FACS apparatus. This enabled determination of precise distribution of Photofrin between malignant tumour cells (IgG negative) and host cells (IgG positive) contained in a murine tumours. The work by Chan *et al.* (1988) has shown that host cells other than macrophages (lymphocytes and polymorphonuclear leukocytes) do not accumulate photosensitiser AlClSPC. It seems reasonable to expect a similar selectivity with Photofrin to be accumulated in macrophages and not in the other host cells, although this remains to be experimentally verified. Since, in addition, most of the host cells are TAM, the IgG positive cells which in this work were shown to have high levels of Photofrin could in fact be identified as macrophages.

The experimental data presented in this work demonstrate that at least up to 4 days after Photofrin administration there is more drug accumulated in IgG positive than in IgG negative cells. Taking into account the content of host cells in SCCVII tumour (~40%), the data indicate that most of the photosensitiser material is in fact contained in TAM, not in the tumour cells. This is particularly pronounced between 4 and 24 h postadministration of Photofrin when there is up to three times more photosensitiser in IgG positive than in IgG negative cells on per cell basis. Based on per cell protein calculation, this ratio is greater than nine for IgG + over IgG − cells.

The measurement of cellular Photofrin content by FACS was verified by an independent measurement of the drug content in sorted cells using chemical extraction of porphyrin followed by a fluorometric assay. This other method, however, includes hydrolysis and disaggregation of porphyrin material, which results in conversion of all photosensitiser components into a more uniform material with optimal fluorescence yield (Korbelik & Hung, 1991; Brown & Vernon, 1990). This is not the case with Photofrin fluorescence measurement in live cells during the FACS sorting, which registers the fluorescence intensity strongly dominated by highly fluorescing monomeric species, and does not reflect the concentration of highly aggregated and less-fluorescening species of this drug (Moan & Sommer, 1983; Brown & Vernon, 1990). The total Photofrin content in the cells not determined by the FACS measurement has thus been assessed by the extraction method.

The comparison of the results obtained with these two methods for Photofrin measurement (Figure 4) does not reveal any major discrepancies, both methods show higher levels of sensitiser in IgG positive compared to IgG negative cells. The only possible difference may be in the time when maximal value for IgG + /IgG − for Photofrin levels is reached. This peak time is observed earlier with the extraction method (4 h postadministration), than with FACS measurement (12 h postadministration); however, this cannot be fully supported by statistical calculation (see Figure 4). This possible difference is not unexpected, since at the highest Photofrin levels, attained 4 h postadministration, much of the drug in the cells could be in highly aggregated form and thus underestimated by the FACS measurement. A few hours later, following intracellular dissociation of its aggregates, Photofrin can reach its strongest fluorescence.

There seem to exist two potential impediments to the FACS method used in this work. The procedure for dispersion of the tumour into single cell suspension takes time (more than 1 h) and exposes cells to digestion enzymes; all of which could result in some loss of photosensitiser from the cells. The other potential problem is photodestruction (photobleaching) of Photofrin by exposure to the strong excitation light (FACS laser). This exposure is, however, very short. Moreover, there are no indications that the rate of photodestruction is different in tumour and host cells under our experimental conditions. In a control experiment, in which TAM were separated from tumour cells by taking advantage of their much more rapid attachment to the plastic substrate, a 4.9 times higher level of Photofrin in TAM enriched population compared to tumour cell fraction was detected (Table I). In this case FACS was not used, and thus there could be no Photofrin photodestruction.

Compared to the TAM population sorted by FACS, the population of TAM selected by the differential attachment procedure may be enriched in activated macrophages. The most likely reason for this difference is that not all macrophages contained in IgG positive tumour fraction would attach to the substrate. The non-attaching TAM, which are probably not in an activated state and thus less active in phagocytising Photofrin than attaching TAM, are lost in the differential attachment procedure. This factor seems most likely to be responsible for a difference in Photofrin levels found in the TAM populations selected by these two techniques.

We have also addressed the concern that the enzymatic dissociation procedure, used for obtaining single cell suspension from tumours could induce a different loss of Photofrin from TAM and tumour cells. The Photofrin loss seen in the experiment designed to examine this issue (Table II) was greater in macrophages than in tumour cells. This suggests that higher Photofrin levels in IgG positive cells compared to IgG negative cells (Figures 2–4) could not be an artefact induced by the enzymatic dissociation procedure. It should be also noted, that the enzymatic treatment (exposure to the cocktail consisting of trypsin, collagenase and DNAase, for 30 min at 37°C) is much harsher to isolated cells attached to the Petri dish substrate (although no cell lysis was detected) than to cells contained in pieces of tumour tissue. The loss of Photofrin from cells during the tumour dissociation is, therefore, probably considerably lower than under conditions of the *in vitro* experiment. In the same experiment, it was shown that *in vitro* peritoneal macrophages and TAM exhibit higher capacity of Photofrin uptake than SCCVII tumour cells (Table II). More detailed analysis of Photofrin uptake and clearance from macrophages and SCCVII tumour cells is reported elsewhere (Korbelik *et al.*, 1991).

The data presented in this study offer clear evidence that in SCCVII murine tumour much higher levels of Photofrin are accumulated in TAM than in tumour cells. This result strongly supports previously published, but less direct evidence and suggestions by a number of investigators (Bugelski *et al.*, 1981; Chan *et al.*, 1988; Jori, 1989; Henderson &

Bellnier, 1989). The high content of TAM in the SCCVII tumour is by no means unusual. There is increasing evidence that many animal and human tumours are characterised by a relatively high macrophage content (e.g. Lindsay *et al.*, 1982; Milas *et al.*, 1987; Eccles & Alexander, 1974; Svennvig & Svaar, 1979). It remains to be verified that a similar pattern of distribution of Photofrin and other photosensitisers between TAM and tumour cells could be found in other tumours. With Lewis lung carcinoma grown on C57B1 mice and using the FACS technique described in this work, we have obtained approximately 3.6 times higher Photofrin levels in IgG positive than in IgG negative cells at 24 h after photosensitiser administration (Korbelik, 1991).

In tumours with high macrophage content the preferential accumulation of Photofrin in TAM could significantly contribute to tumour localisation of Photofrin in tumour tissue. The fact that most of photosensitiser material could be accumulated in TAM population has obviously important implications for PDT. Light energy dependent production of TNF by murine macrophages has been demonstrated after *in vitro* PDT using Photofrin (Evans *et al.*, 1990). Release of large amounts of prostaglandin E from peritoneal murine macrophages after Photofrin-based PDT has also been reported (Henderson & Donovan, 1989). There are strong indications that PDT-induced immunosuppression (Lynch *et al.*, 1989) and lethality due to TNF-induced cahexia following PDT are mediated by macrophages (Ferrario & Gomer, 1990; Darling *et al.*, 1990).

The contribution of Dr R.E. Durand in evaluation of the FACS technique is gratefully acknowledged. Technical assistance from Mrs N. LePard (FACS operation) and Mrs S. Mills (tumour maintenance) is also acknowledged and appreciated. This work was supported by operating and scholarship grants awarded by B.C. Health Care Research Foundation.

### References

BROWN, S.B. & VERNON, D.I. (1990). The quantitative determination of porphyrins in tissues and body fluids: applications in studies of photodynamic therapy. In *Photodynamic Therapy of Neoplastic Disease* (Vol. I), Kessel, D. (ed.), p. 109, CRC Press: Boca Raton.

BUGELSKI, P.J., PORTER, C.W. & DOUGHERTY, T.J. (1981). Autoradiographic distribution of hematoporphyrin derivative in normal and tumor tissue of the mouse. *Cancer Res.*, **41**, 4606.

CHAN, W.-S., MARSHALL, J.F., LAM, G.Y.F. & HART, I.R. (1988). Tissue uptake, distribution and potency of the photoactivable dye chloroaluminum sulfonated phthalocyanine in mice bearing transplatable tumors. *Cancer Res.*, **48**, 3040.

CHAPLIN, D.J., OLIVE, P.L. & DURAND, R.E. (1987). Intermittent blood flow in a murine tumor: radiobiological effects. *Cancer Res.*, **47**, 597.

DARLING, G., FRAKER, D.L., JENSEN, J.C., GORSCHBOTH, C.M. & NORTON, J.A. (1990). Cachectic effects of recombinant human tumor necrosis factor in rats. *Cancer Res.*, **50**, 4008.

ECCLES, S.A. & ALEXANDER, P. (1974). Macrophages content of tumors in relation to metastic spread and host immune reaction. *Nature*, **250**, 667.

EVANS, S., MATTHEWS, W., PERRY, R., FRAKER, D., NORTON, J. & PASS, H.I. (1990). Effect of photodynamic therapy on tumor necrosis factor production by murine macrophages. *J. Natl Cancer Inst.*, **82**, 34.

FERRARIO, A. & GOMER, C.J. (1990). Systemic toxicity in mice induced by localized porphyrin photodynamic therapy. *Cancer Res.*, **50**, 539.

HENDERSON, B.W. & BELLNIER, D.A. (1989). Tissue localization of photosensitizers and the mechanism of photodynamic tissue destruction. *Ciba Found. Symp.*, **146**, 112.

HENDERSON, B.W. & DONOVAN, J.M. (1989). Release of prostaglandin $E_2$ from cells by photodynamic treatment *in vitro*. *Cancer Res.*, **49**, 6896.

JORI, G. (1989). *In vivo* transport and pharmacokinetic behaviour of tumour photosensitizers. *Ciba Found. Symp.*, **146**, 78.

KORBELIK, M. (1991). Photofrin and other photosensitizers for use in photodynamic therapy of cancer. *Period. Biol.* (in press).

KORBELIK, M. & HUNG, J. (1991). Cellular delivery and retention of Photofrin II: the effects of interaction with human plasma proteins. *Photochem. Photobiol.*, **53**, 501.

KORBELIK, M., KROSL, G. & CHAPLIN, D.J. (1991). Photofrin uptake by murine macrophages. *Cancer Res.*, **51**, 2251.

LINDSAY, J.M., MANNING, L. & WOOD, G.W. (1982). Exclusive binding of immunoglobulin to Fcγ receptors on macrophages in 3-methylcholantrene-induced tumors. *J. Natl Cancer Inst.*, **69**, 1163.

LYNCH, D.H., HADDAD, S., KING, V.J., OTT, M.J., STRAIGHT, R.C. & JOLLES, C.J. (1989). Systemic immunosuppression induced by photodynamic therapy (PDT) is adoptively transferred by macrophages. *Photochem. Photobiol.*, **49**, 453.

MILAS, L., WIKE, J., HUNTER, N., VOLPE, J. & BASIC, I. (1987). Macrophage content of murine sarcomas and carcinomas: association with tumor growth parameters and radiocurability. *Cancer Res.*, **47**, 1069.

MOAN, J. & SOMMER, S. (1983). Uptake of the components of hematoporphyrin derivative by cells and tumors. *Cancer Lett.*, **21**, 167.

OLIVE, P.L. (1989). Distribution, oxygenation, and clonogenicity of macrophages in a murine tumor. *Cancer Commun.*, **1**, 93.

RUSSELL, S.W., GILLESPIE, G.Y. & PACE, J.L. (1980). Evidence for mononuclear phagocytes in solid neoplasms and appraisal of their non-specific cytotoxic capabilities. *Contemp. Top. Immunobiol.*, **10**, 143.

SVENNVIG, J.L. & SVAAR, H. (1979). Content and distribution of macrophages and lymphocytes in solid malignant human tumors. *Int. J. Cancer*, **24**, 754.

WOOD, G.W. & GOLLAHON, K. (1977). Detection and quantitation of macrophage infiltration into primary human tumors with the use of cell-surface markers. *J. Natl Cancer Inst.*, **59**, 1081.